# Sensitive Periods, Vasotocin-Family Peptides, and the Evolution and Development of Social Behavior

**DOI:** 10.3389/fendo.2017.00189

**Published:** 2017-08-02

**Authors:** Nicole M. Baran

**Affiliations:** ^1^Department of Psychology, Cornell University, Ithaca, NY, United States; ^2^School of Biological Sciences, Georgia Institute of Technology, Atlanta, GA, United States

**Keywords:** evo-devo, vasopressin, nonapeptides, sensitive periods, social behavior, vocal learning, developmental psychobiology

## Abstract

Nonapeptides, by modulating the activity of neural circuits in specific social contexts, provide an important mechanism underlying the evolution of diverse behavioral phenotypes across vertebrate taxa. Vasotocin-family nonapeptides, in particular, have been found to be involved in behavioral plasticity and diversity in social behavior, including seasonal variation, sexual dimorphism, and species differences. Although nonapeptides have been the focus of a great deal of research over the last several decades, the vast majority of this work has focused on adults. However, behavioral diversity may also be explained by the ways in which these peptides shape neural circuits and influence social processes during development. In this review, I synthesize comparative work on vasotocin-family peptides during development and classic work on early forms of social learning in developmental psychobiology. I also summarize recent work demonstrating that early life manipulations of the nonapeptide system alter attachment, affiliation, and vocal learning in zebra finches. I thus hypothesize that vasotocin-family peptides are involved in the evolution of social behaviors through their influence on learning during sensitive periods in social development.

## Introduction

Both extrinsic and intrinsic experience broadly shape the functional organization of the brain. Functional maturation critically depends on input at particular points in time to ensure that nervous systems are organized to enable the organism to survive and reproduce in maturity. The basic plan of the brain is, of course, governed by genetic expression. However, its development is influenced at every point along the way by the “environment,” broadly construed (1–5).

The development of the brain is shaped by experience that comes from outside the organism, but also by chemical signals that are generated by the organism itself. These signals include chemical gradients that guide the growth of neuronal projections to their targets, or longer distance chemical messengers that modulate the activity of complex neural circuits. Importantly, chemical messengers, such as steroid hormones or neuropeptides, provide a signal to coordinate the development of multiple tissues. Thus, there are important parallels between the organizational role that hormones play during development (intrinsic experience) and the organizational effects of experience that comes from outside of the organism (extrinsic experience).

There is, however, little research investigating the mechanisms that govern how organisms are so exquisitely sensitive to particular kinds of sensory input at specific points in development. In particular, there is a significant gap in our understanding of the neural and neuroendocrine mechanisms that modulate sensitive periods in development, even for potent forms of early learning, such as imprinting or vocal learning. What mechanisms shape the timing, existence, and nature of these sensitive periods in development, particularly given the significant impact that early social learning has on adult phenotype?

The nonapeptides, which provide an evolvable mechanism for modulating the activity of whole neural circuits in specific social contexts, may play an important role in the evolution and development of social phenotypes across vertebrate taxa. The majority of nonapeptide research in the last several decades has focused on adult organisms. Fortunately, there is a re-emerging interest in the effects of the nonapeptides during development (6, 7). The hypothesis that the nonapeptides may exert organizational effects on the brain by producing long term or permanent changes in neural structure was first proposed during the 1980s [arginine vasopressin (AVP) (8); oxytocin (OT) (9)]. Despite several intriguing findings which suggested that the nonapeptides are important during development, there was relatively little interest in this area until recently. This resurgence was largely driven by the realization that exogenous administration of synthetic OT to induce labor may have unknown side effects at a critical point in brain development (10, 11). Furthermore, it was realized that the nonapeptides may also have potential relevance to understanding social deficit and neurodevelopmental disorders (12).

Here, I argue that the nonapeptides, particularly in the vasotocin family, play a critical role during sensitive periods for social learning. Nonapeptides are known to influence many brain regions involved in multiple social behaviors. I present some speculative evidence that they may also do so during development in ways that have profound consequences for adult social behavior. Similar to discoveries in the newly emerging field of evolutionary developmental biology (evo-devo), this evidence suggests that subtle variations in nonapeptide circuitry during development may underlie species differences in social behaviors through their influence on social learning processes. To frame this argument, I attempt to link findings from several previously unconnected fields, including classical work in developmental psychobiology on sensitive periods, the evolution and development of the nonapeptide system, and several theories about how nonapeptides affect social processes. Finally, I will summarize recent work in the zebra finch (*Taeniopygia guttata*) supporting this claim which demonstrates that early life manipulations of the nonapeptide system alter attachment to parents, affiliative behavior in adult males, and vocal learning.

## Sensitive Periods in Development

### What Is a Sensitive Period?

There is extensive evidence of early sensitive periods in development, in which an organism demonstrates a marked susceptibility or vulnerability to particular stimuli during a limited time window early in life (13, 14). This phenomenon reflects a developmental phase of built-in competence for exchange between an organism and its environment. Sensitive periods are most often observed in the context of what is known as experience-expectant learning, where an organism depends on certain types of experience in order to develop normally. By experimentally removing or altering the stimulation, we can reveal the extent to which an organism requires the species-typical input for species-typical development. For example, visual deprivation early in life in a number of species can cause disorganization of the cortical columns necessary to process visual stimuli (15). In addition, certain cues in an organism’s environment may provide information about which phenotypes will be most successful given the environment the organism is likely to encounter. In this case, developing organisms “sample” their environment during sensitive periods for cues which direct development in an adaptive direction. For example, early nutritional stress can serve to program physiological function in ways that would enhance postnatal survival under conditions of intermittent or poor nutrition (16).

Some of the most striking examples of sensitive periods in development occur in the social domain. One particularly potent form of an early sensitive period is imprinting. Imprinting, such as filial or sexual imprinting, is defined as a form of learning that (1) can only take place during a restricted window of time in an individual’s life, (2) is irreversible, (3) involves the learning of species specific or individual-specific characters, and (4) may occur at a time when the appropriate behavior itself is not yet performed (17). Visual imprinting phenomena have been best studied in birds (18–23), where the circuit underlying filial imprinting has been well characterized (24–26). Precocial birds, such as domestic chickens and ducks, will approach and follow any object which they were exposed to immediately after hatching, typically the mother. In addition, there is work from a number of fish species demonstrating imprinting phenomena used for species and kin recognition, primarily in the olfactory domain (27–34).

There are also more subtle forms of sensitive periods in social development, in which experience with caregivers early in life shapes later social relationships (35–38). Across taxa, isolation from conspecifics and caregivers results in significant disruptions to social functioning later in life (39–42). Furthermore, research in rats has demonstrated that early experiences of maternal care (e.g., licking and grooming behaviors) can alter both the responsiveness to stressors and maternal behavior in adulthood (37, 43, 44). Song learning in birds and vocal learning in humans, though they occur later in development, are also examples of sensitive periods in development in which the developing organism is dependent on interactions with adult caregivers to learn species-typical vocal structures (45, 46).

What appears to be common across these different kinds of sensitive periods in development is competitive exclusion—a particular class of sensory input from the environment is particularly influential, to the exclusion of others (47). These early forms of social learning, particularly about the identity, features, and valence of caregivers, provides an important foundation for later learning. Both learning the characteristics of and maintaining the motivation to be proximal to caregivers provide developing organisms with food and protection but also an abundance of opportunities for social learning. Indeed, these early social experiences provide the foundation upon which all future social interactions are built.

In each of these cases, we have a sense of what circuits are involved in the sensitive periods (as well as a number of ways to disrupt those circuits), but critically we do not know the mechanisms of how or why there is a particular kind of sensitivity in the first place. For example, the filial imprinting work in birds has focused on forebrain regions thought to be homologous to mammalian cortex which are involved in visual processing and multimodal association (24–26, 48). But why those particular inputs at that particular time?

### Established Mechanisms Underlying Sensitive Periods

One theory about how sensitive periods emerge is based on the observation that the sensory systems do not all become functional at the same time, but rather sequentially. In both birds and mammals, the first sensory modality to become functional is the tactile/vestibular system, followed in order by the chemosensory/olfactory, auditory, and visual systems (49, 50). Based on the pioneering work of Gilbert Gottlieb in avian development, Turkewitz and Kenny proposed that the invariance in the sequential onset of sensory function results in a reduction in the complexity of the sensory experience for the developing organism and a more reliable structure to the prenatal and early postnatal experience (51). Developing organisms are not bombarded with novel stimuli in all sensory modalities upon birth, but instead encounter a drastically reduced sensory experience. Earlier-developing systems (i.e., tactile and olfactory) in fact develop under reduced competition from other sensory modalities. This allows later-developing systems (i.e., visual and auditory) to build on associations formed in earlier-developing systems.

Furthermore, given that many sensory systems begin to develop prior to birth or hatching, the *in utero* and *in ovo* environments provide learning opportunities for the developing embryo. Precocial birds, which are born with all sensory systems functional at birth, benefit from extensive learning that takes place in the egg which they use to support the “emergence, maintenance, and transformation of behavior” (52). For example, in ducks, the preference for the maternal assembly call is dependent upon the prenatal exposure of the embryo either to its own vocalizations or those of its siblings in the days prior to hatching (53). Similarly, bob white quail denied interaction with broodmates after hatching fail to develop preferences for species-specific maternal cues (54). Even altricial rodents, which are less mature at birth, also use olfactory associations formed *in utero* to perform suckling behaviors (55).

In addition, generalized physiological arousal has been identified as a critical component of a young organism’s perceptual learning and development. In human infants, for example, there is a strong association between arousal levels and sensitivities to sensory stimulation (56–58). Physiological arousal can be manipulated neurochemically, or by simply making sensory stimuli more salient. For example, only rat pups receiving either tactile stimulation or injected with amphetamine while exposed to an artificial odor preferred to suckle nipples coated in the familiar odor (59). Furthermore, this process can be disrupted by a poorly timed change in arousal state. Injection of norepinephrine into quail embryos in the absence of exposure to appropriate auditory stimulation resulted in disrupted preference for the familiar maternal call (60). This work suggests that normal social development depends on physiological systems that mediate arousal and attention in the appropriate social environments early in life.

## Neuroendocrine Signals as a Potential Mechanism Underlying Sensitive Periods

I propose that neuroendocrine mechanisms are also prime candidates for mediating sensitive periods in development. Hormones, which are typically defined as long-distance chemical signals, act directly on the cellular processes of neurons, but they also affect more general physiological systems, such as arousal, gonadal state, and metabolic function. Hormones influence multiple tissues simultaneously and modulate physiological and developmental processes across a wide spatial and temporal distance (61). This enables organisms to simultaneously coordinate many tissues or recruit whole neural circuits for an important task (62). Indeed, hormonal signals can provide a functional link between otherwise unconnected neuronal populations (63).

Most of the developmental effects of hormones have been studied in the context of steroid hormones and sexual differentiation (64–68). The focus of this work has been on how the hormones directly affect cellular function and the connectivity of neural circuits. However, many neuroendocrine signals have the potential to play a role in the organization of the social brain specifically by altering learning processes. Glucocorticoids, sex steroids, and neuropeptides have all been shown to be involved in learning and memory, both directly and indirectly (69). Nevertheless, there remains a gap in our understanding the role that such signals play in influencing the outcome of development in the context of important social experiences. Furthermore, the diversity of social phenotypes both within and between species begs the question as to how the unique features of both the organism’s early social experiences, as well as evolved differences in their neuroendocrine function, support the evolution and development of novel social phenotypes. For the purposes of this review, I focus on vasotocin-family neuropeptides, but many of the general principles of my argument may apply to other neuroendocrine signals, as well.

### Overview of the Nonapeptides

Over the last several decades, much research effort has been devoted to vasotocin family of neuropeptides (i.e., nonapeptides), which includes [arginine vasotocin (AVT), found in non-mammals and likely the ancestral peptide] and its mammalian homolog AVP; and the OT-like peptides [isotocin (IT), found in fish, mesotocin (MT), found in lung fish and non-eutherian tetrapods, including birds; and OT, found in mammals] (70, 71). The nonapeptides derive from an evolutionarily ancient neuromodulator. In the earliest vertebrates, only one gene was present (AVT), but sometime after *Agnatha* (lampreys and hagfish) a gene duplication event led to the divergence of the vasotocin (AVT/AVP) and OT (IT/MT/OT) lineages (72). Although these two lineages differ in only a single amino acid, the nonapeptides appear to have evolved quite distinct functions.

This is because the nonapeptides coevolved with their receptors, which are classic G-protein coupled receptors. There are typically four receptor subtypes for the nonapeptides within each species: V1a, V1b, V2, and OT (VT4, VT1, VT2, and VT3, respectively, in birds). The amino acid sequences of each receptor subtypes are more similar to each other across species (~90%) than they are to different subtypes within a single species (~45%) (73). When binding to their receptors, nonapeptides can have a multitude of effects on neurons, including changes to gene transcription, recruitment of intracellular calcium, neuroprotective effects, and alterations to long-term potentiation mechanisms (74). The sequencing of the vertebrate nonapeptide receptor genes suggests that the core–ligand receptor interaction sites have remained remarkably conserved, while varying the intracellular components, and thus their downstream effects (75). Receptors for nonapeptides are distributed throughout the brain, but importantly, the distribution of each of the receptor subtypes can vary widely by species, sex, age, and social context (76–78).

The primary sources of nonapeptides are the AVP/OT cell groups of the supraoptic nucleus (SON) and paraventricular nucleus (PVN) nuclei of the hypothalamus, as well as smaller extra-hypothalamic accessory cell groups, including the medial amygdala (MeA), medial bed nucleus of the stria terminalis (BSTm), lateral septum (LS), olfactory bulb (OB), and suprachiasmatic nucleus (SCN) (79, 80). The production of AVT/AVP, particularly in from the extra-hypothalamic cell groups, is often sexually dimorphic (usually male greater than female), organized by sex steroids during development, and sensitive to changes in gonadal state (81–88).

In order to understand the modulatory role of AVT/AVP during development, we need to consider the sources of the peptide, the sites of action in the body, and the functional consequences. There are three primary physiological systems influenced by AVT/AVP. The first can be summarized as AVT/AVP’s involvement in vasoconstriction and water balance. AVT/AVP, when released by magnocellular neurons in the PVN and SON of the hypothalamus, is released into the posterior pituitary. From there, AVT/AVP enters general circulation where exerts antidiuretic effects throughout the body.

The second is AVT/AVP’s involvement in the stress response. AVT/AVP, when it is released from the anterior pituitary by parvocellular neurons in the PVN, is at the top of the hypothalamic–pituitary–adrenal (HPA) axis. The HPA axis regulates the physiological response to stressors, helping the body mobilize resources in response to challenges in its environment. AVT/AVP, along with corticotropic releasing factor (CRF) serves as a releasing hormone for adrenocorticotropin-releasing hormone (ACTH) from the anterior pituitary, which is the chemical signal that leads to the release of glucocorticoids from adrenal tissue (89). AVT/AVP is not itself the major releasing hormone for ACTH, but it plays a critical role by potentiating the biological activity of CRF (90). Parvocellular AVT/AVP neurons are highly responsive to stress (91). Acute stress increases the production of both CRF and AVT/AVP in the PVN (92, 93).

Finally, multiple cell groups in the brain contribute to the central pool of nonapeptides with highly diverse functional consequences. Some of the same neurons that project into the pituitary also send projections back into the brain. In addition, the extra-hypothalamic accessory cell groups, including the MeA, BSTm, LS, OB, and SCN, contribute to the central pool of peptides (79, 80). Each of the AVT/AVP cell groups has a different pattern of activity and neural release, which is ultimately a function of the kinds of computation those neurons perform (87). Variation in how the nonapeptides affect the interconnected set of brain nuclei known as the social behavior network and other brain regions is thought to underlie the multiple effects of AVT/AVP across species (94, 95). Indeed, nonapeptides have been implicated in species differences in many diverse social behavioral domains (76, 77, 96–104).

### Nonapeptides in Development

The vast majority of this comparative work, however, has focused on nonapeptide function in adulthood. We in fact understand remarkably little about how nonapeptides shape social behaviors during development, particularly those behaviors for which plasticity, flexibility, and learning are critical. The effect that nonapeptides have on social learning in each species is influenced by when they act relative to important social experiences and in what brain regions. Across the few vertebrate species in which it has been investigated, the sequence of nonapeptide cell group maturation appears to be conserved. The first AVT/AVP immunostaining is consistently found in the SON followed by the PVN (105–112). In tetrapods, this is followed by production in the extra-hypothalamic cell groups, such as the BSTm and MeA, which exhibit steroid hormone-mediated sexual dimorphism in AVT/AVP staining (81, 113–115).

The most detailed developmental work in the nonapeptide system comes from rodents, particularly the rat. In rats, the neurons of the SON and PVN have formed before birth by 12–14 days postfertilization (dpf), gestation is 21 days in the rat (108). In the rat brain, the first AVP staining is observed between 14–18 dpf, which steadily increases to adult levels by postnatal day 30 (108, 113). Between birth and postnatal day 21, there is a 22- to 30-fold increase in AVP production by the pituitary, suggesting that the neurons that project from the hypothalamus to the pituitary are gradually coming on-line during development. By contrast, the cell groups of the BSTm and MeA show AVP staining only after birth, with the MeA delayed relative to the BSTm. AVP mRNA was only observed in the BSTm on postnatal day 3 and in the MeA on day 5 in male rats and day 14 and day 35, respectively, in female rats (113). The levels of AVP, thus, reach adult levels by postnatal day 35 in the BSTm and day 60 in the MeA in both sexes (113).

A similar developmental trajectory is found in the domestic chicken, despite substantial evolutionary distance and its precocial development. AVT is observed early in development in the chicken embryo SON and PVN, as early as 6 dpf (109–112). AVT is detectable in the BTSm by 12 dpf, which increases until hatching at 17 dpf before dropping precipitously in days after hatching (114). AVT then increases gradually in males until 129 dpf (114). Thus, the onset of function of all the cell groups in the SON, PVN, and BTSm occur while the chicken is still in the egg.

Very limited information on the development of these cell groups exists from other species, but the sequential development of the hypothalamic cell groups appears similar. In humans, AVP is detectable in the SON and PVN at 77 and 91 dpf, respectively (107, 116). Thus, in humans, hypothalamic production of AVP begins before birth. Even in zebra fish, two cell groups in the rostral diencephalon and hypothalamic regions show AVT mRNA expression sequentially starting at 24 h postfertilization (106). The embryonic development of the nonapeptide system has not been explored in songbirds. An early paper that explored the development of the nonapeptide circuitry in canaries found that AVT was expressed in the PVN at 4 weeks, but no staining was observed in the BSTm or LS until later at 8–12 weeks of age (117).

**Figure 1:**
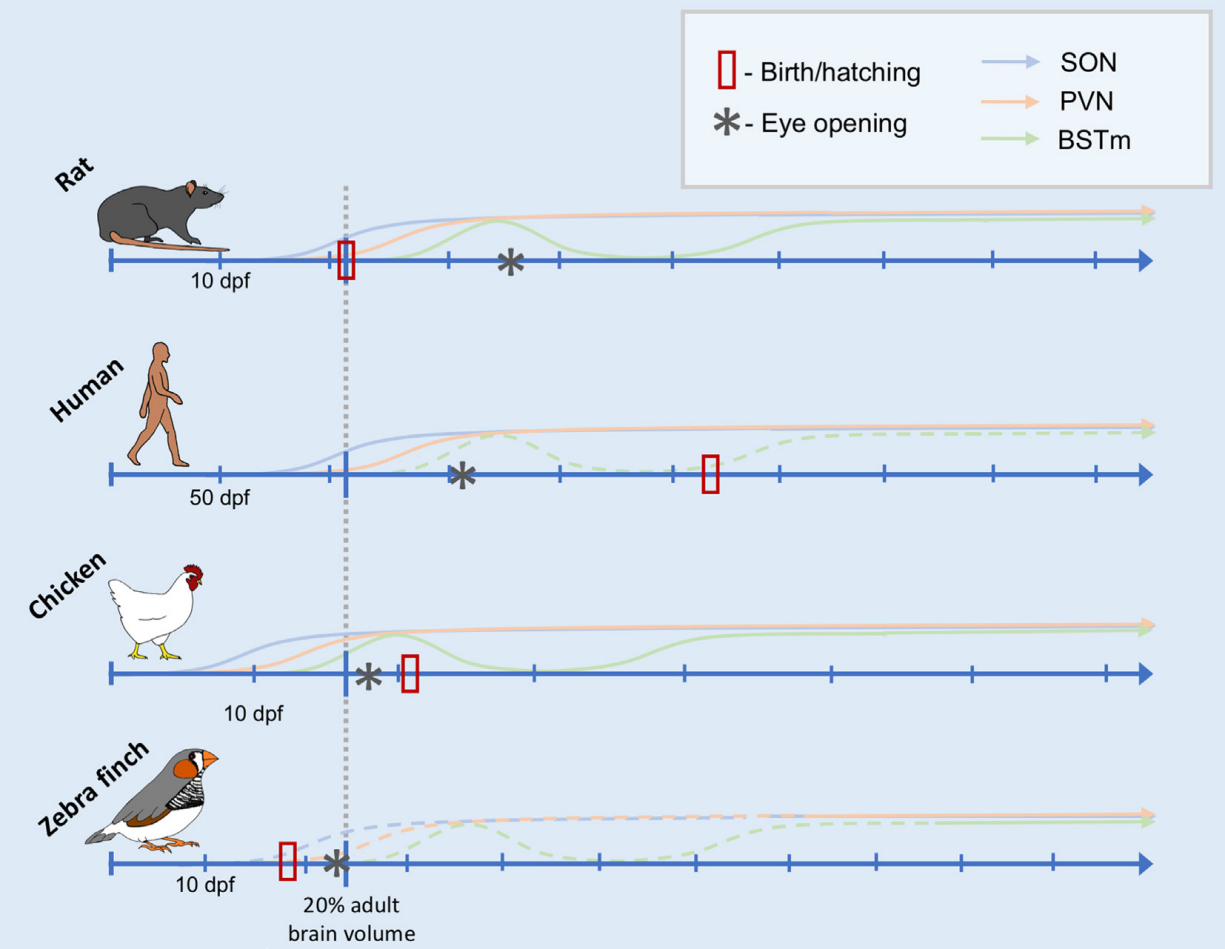
Comparative timeline of arginine vasotocin (AVT)/arginine vasopressin (AVP) cell group development. Conceptual timeline illustrating the production of AVT/AVP across early development in rats, humans, chickens, and zebra finches in three main AVT/AVP cell groups: the supraoptic nucleus (SON, blue), paraventricular nucleus (PVN) of the hypothalamus (PVN, orange), and the medial bed nucleus of the stria terminalis (BSTm, green). The *x*-axis of the timeline is scaled to the neurodevelopmental time point of when the organism is estimated to reach 20% total adult brain volume (indicated by the gray dotted line intersecting each timeline). Days postfertilization (dpf) are indicated by hatchmarks along each timeline. Solid lines indicate solid data (see text for references), whereas dotted lines are predicted results. The data from which this illustration is created include counts of AVT/AVP immunoreactive cells, mRNA expression, and peptide concentrations (see Table 1). Thus, the *y*-axis does not have a scale, as it is not clear how these different data types are comparable across species, cell group, study, etc. The date of hatching or birth for each species is indicated by a red rectangle. Date of eye opening is indicated by an asterisk.

Taken together, these data suggest that the relative timing of the onset of function of the AVT/AVP cell groups is, in fact, remarkably conserved throughout evolution. Furthermore, the sequence of general neurodevelopmental events (from neurogenesis to eye opening) is also very predictable (118). What changes more is the timing of birth or hatching and, thus, early social experiences relative to these ontological changes. Inspired by Workman et al., Figure 1 depicts the development of the nonapeptide system scaled according to when the brain reaches 20% total brain volume, a milestone that is highly correlated with other neurodevelopmental events (118). This is based on data from three species for which we have some information about the development of the nonapeptide system: rats, humans (SON and PVN only), and chickens. The fact that neurodevelopmental events occur in a highly stereotyped sequence allows us to make predictions about the maturation of the nonapeptide system for cell groups or other species for which we do not have data, as well. Figure 1 shows the predicted timing of AVT synthesis in the respective cell groups in the zebra finch brain, based on general data on zebra finch neurodevelopment. Data supporting the figure can be found in Tables 1 and 2.

**Table 1:** Earliest day postfertilization of arginine vasotocin (AVT)/arginine vasopressin labeling found.

Species	SON	PVN	BSTm	MeA	Reference
Rat	16	18	24♂, 35♀	25♂, 56♀	(105, 108, 113, 119, 120)
	IHC, RIA	IHC, RIA	ISH	ISH	

Human	77	91			(107, 116)
	IHC	IHC			

Chicken	6	7.5	14♂, 16♀		(109–112, 114, 115)
	IHC, RIA	IHC, RIA	IHC		

*The type of evidence for each time point is indicated below. If available, we use data from the labeling of the AVT peptide, from either immunohistochemistry (IHC) labeling cell bodies and radioimmunoassay (RIA) from brain and pituitary. In some cases, only data from *in situ* hybridization (ISH) labeling AVT gene expression is available. Sex differences are indicated, where noted. Cell group abbreviations: supraoptic nucleus (SON) of the hypothalamus, paraventricular nucleus (PVN) of the hypothalamus, medial bed nucleus of the stria terminalis (BSTm), and medial amygdala (MeA)*.

**Table 2:** Data are presented for 20% brain volume, eye opening, amygdala neurogenesis peak, and the date of birth or hatching.

Species	Neurodevelopmental event	DPF	Reference
Rat	20% brain volume	19*	(118)
	Eye opening	36	(121)
	Amygdala neurogenesis peak	15	(122)
	Birth/hatching	21	

Human	20% brain volume	118*	(118)
	Eye opening	157.5	(123)
	Amygdala neurogenesis peak	46*	(118)
	Birth/hatching	270	

Chicken	20% brain volume	17	(124)
	Eye opening	18	(125)
	Amygdala neurogenesis peak		
	Birth/hatching	21	

Zebra finch	20% brain volume	24	(126)
	Eye opening	23	(127)
	Amygdala neurogenesis peak	30	(126)
	Birth/hatching	18	

*Asterisks indicate that the date is predicted based on the model in Ref. (118)*.

*DPF, days postfertilization*.

A few notes of caution are required. First, it is important to note that these predictions are still quite speculative, given the available data. The Workman et al. model has not been applied to avian systems, so the extent to which we can extrapolate the findings to chickens and zebra finches is still unknown. More information about when key neurodevelopmental events occur in the zebra finch brain may also help to determine whether the maturation of the AVT system would be more similar to the altricial but distantly related rat or the precocial but more closely related chicken.

Second, Figure 1 was created using available data, which is a mix of immunohistochemistry (IHC) labeling of AVT or AVP protein and *in situ* hybridization (ISH) labeling AVT/AVP gene expression (see Table 1). It is also based on labeling of these in the cell bodies within the respective brain regions. AVT/AVP is first synthesized as a large protein precursor molecule, which is enzymatically cleaved into the active hormone (80, 128). The active hormone must then be packaged into specialized neurosecretory vesicles and transported to the nerve terminals where it is released. Thus, the presence of either ISH or IHC labeling in the cell bodies is not a definitive indicator that the hormone is being released, particularly during development (105).

Third, we have limited information about where nonapeptides are acting during development. Binding sites have been found in the developing mouse and rat brain in both the amygdala and septum between postnatal days 0 and 8, as well as several brain regions where AVP receptors are not expressed in adulthood, including the hippocampus, dentate gyrus, and caudate nucleus (129, 130). In rats, many brain regions also show significant differences in between juveniles and adult (131). The consequences of these brain-region specific changes in receptor expression across development are almost certainly important, but have proven difficult to explore experimentally.

Finally, sex differences in both AVT/AVP production and receptor expression also likely influence developmental processes. For example, there is a delay in AVT/AVP synthesis in females relative to males in the BSTm and MeA (113, 115). Given these differences, we might predict that males would be more affected by AVT/AVP manipulations or by manipulations earlier in development, as compared to females. Some sex differences in receptor expression have also been found, but we have a poor understanding of the functional consequences of these differences.

Nevertheless, manipulations of nonapeptides in rodents very early in life provide evidence that nonapeptides matter in development (6, 7). For example, vasopressin–deficient Brattleboro rat pups show hyperactivity, reduced huddling, and reduced proximity to other pups in the nest compared to wild-type rats (132). Wild-type rat pups treated with a nine-day exposure to AVP showed increased emotionality, activity levels, and grooming in an open field test as juveniles, as well as smaller overall brain size (133). Acute central administration of AVP in wild-type neonatal rat pups was found to decrease the number of ultrasonic vocalizations and reduced locomotor activity in a maternal isolation test (134). In juvenile male rats, both targeted infusion of AVP into the LS and intracerebroventricular infusion increased preference for investigating novel individuals, whereas a V1aR antagonist increased the preference for investigating familiar individuals (135). In addition, V1aR blockade in the LS increased social play behavior in males and decreased it in females, but only when it tested in a familiar environment (136, 137). Neonatal manipulation of AVT or OT in the socially monogamous prairie vole, leads to sex-specific changes in nonapeptide binding in several brain regions in adults and alterations to social behaviors (138–141). For example, sexually naïve males who were treated with AVP early in life were more aggressive than control males but females were less responsive to AVP treatment (140). It is unclear whether the developmental effects of these nonapeptide manipulations are mediated through physiological effects on the body versus binding within the brain. Nevertheless, these studies provide intriguing evidence that the nonapeptides are involved in the development of social behavior.

## Conservation and Novelty

Several researchers have proposed that one way to understand the outsize role of neuropeptides such as AVT/AVP in the evolution of behavioral diversity is through studying the differential expression of the peptides and their receptors (142, 143). The evolution of neuropeptide signaling systems may be highly constrained within the nervous system because mutations altering the proteins themselves would have deleterious effects on the receptor–ligand interactions. However, tweaks to where and in what quantities the peptides and their receptors are expressed via changes to gene regulation provide opportunities to modify the activity of neural circuits (142). Indeed, a number of examples have been identified. For example, variation in the production of AVT has been associated with species differences in social behaviors in birds (144). The number of IT-producing cells in the POA has been associated with cooperative breeding in cichlid fish (103). And the expression of V1aR is associated with variation in mating behaviors in voles (76, 145, 146). However, perhaps an important mechanism underlying this behavioral diversity across species is not just where but also *when* nonapeptides are acting during development.

### Sensorimotor Processing Hypothesis

Much of the research for the role of nonapeptides in social behavior has focused on brain regions with more generalized roles in social processing. However, the “compartmentalization” of AVT/AVP function can extend to include the modulation of species-specific behavioral circuits throughout the central nervous system (142, 147). In their sensorimotor hypothesis, Rose and Moore posit that AVT/AVP can act on sensory pathways to modulate the responsiveness of neurons to particular kinds of sensory stimuli as well as act on motor pathways to modulate behavioral output (147). In this case, the nonapeptide signal is directly modulating the specific circuits that are necessary for the production of the behavior. This theory emerged from comparative work which suggests that AVT modulates the activity of neurons in each step of a sensorimotor processing circuit which controls a complex courtship behavior in male newts (*Taricha granulosa*) (147). In these newts, AVT enhances the highly stereotyped sexual behavior, in which the male embraces the female with all four limbs to induce receptivity (148). AVT enhances this behavior by modulating sensory processing in the visual and olfactory domains as well as motor output at the level of the spinal cord (149–151).

A parallel story may also be true in the case of complex learned behaviors, such as bird song. Interestingly, there is limited evidence that anterior forebrain song learning pathway is sensitive to AVT, at least in adults AVT (88, 152–154). However, each step in the circuit controlling the expression of vocal behavior in birds appears to be partially modulated by AVT (152, 154, 155). Several auditory structures in the forebrain, including the caudomedial mesopallium and the caudomedial nidopallium, highly express V1aR in zebra finches (154). The robust nucleus of the arcopallium (RA, homologous to laryngeal motor cortex) exhibits limited receptor expression, but two nuclei involved in the motor pathway of song production contain AVT receptors. There is AVT immunoreactivity and binding in the intercollicular nucleus (ICo, a region implicated in vocal control) in several songbird species (152, 153, 156, 157). In addition, the key motor nucleus, nXIIts, which innervates the syrinx and is considered part of the song system, contains high levels of mRNA for all three subtypes of AVT receptor (154). However, a sensorimotor account of the role played by these regions during song learning remains to be tested.

### Social Gating Hypothesis

Evolutionary novelty in behavior may also arise when new structures or circuits are modulated in new ways starting early in development. Syal and Finlay claim that what is necessary for the evolution of novel behaviors is changes to how the sensory and motor circuits are attached to the socio-motivational circuitry during early social interactions with caregivers, family, and conspecifics (158). In fact, they view the reciprocally connected network of brain nuclei known as the social behavior network (which includes the major nonapeptide cell groups), as the conserved neural structure that assembles the relevant sensory dimensions of a representation of other individuals (i.e., caregivers, mating partners, rivals) and attaches that representation to motivations and actions appropriate to their social context (158). The social behavior network is highly connected to the mesolimbic reward system via the BSTm, MeA, and LS, which all contain nonapeptide cell groups. Other structures commonly associated with reward and motivation, such as the ventral tegmental area, ventral pallidum, and nucleus accumbens, also densely express receptors for nonapeptides (159).

In this context, the modulatory signal produced by the nonapeptide cell groups, by acting on receptors throughout the brain, can be used to bias attention toward certain kinds of sensory stimuli or to reward the performance of certain behaviors. Consequently, it is easy to imagine how even tiny tweaks to the system, such as gene mutations that change the regulation of a receptor gene or slightly alter its downstream functions, might have large effects on whole neural circuits. Thus, the nonapeptide system may provide a mechanism whereby evolution generates novel social behavior using an otherwise highly conserved brain.

The effect of changes to the nonapeptide system would, thus, be expected to be even more consequential in development, particularly when coupled with salient social experiences. If indeed nonapeptides are gating social learning, then the nonapeptides may function by biasing a young organisms’ attention toward the behaviors exhibited by their family or other socially relevant conspecifics. For example, a primary reason why the development of the nonapeptide system may underlie important social development is because of its central role in olfactory processing. Early social experiences are often highly olfactory and thermotactile in nature. For example, suckling behavior in rat pups is dependent upon odor processing in the accessory olfactory system and MeA, which allows them to learn the odor of their mother’s amniotic fluid and of the saliva of their broodmates to guide nipple attachment (160). Even zebra finches, which do not have an accessory olfactory system and which are thought to be more responsive to auditory and visual stimuli, show olfactory preferences for their natal nest (161). Thus, early olfactory experiences provide some of the first forms of social learning about conspecifics at the same time that the relevant behavioral circuits begin responding to nonapeptides.

Increased attention to relevant social stimuli would provide opportunities for social learning, which could also be reinforced by socio-motivational circuits. Early sensitivity to social stimuli would support future social learning, leading to accumulating effects. On the other hand, genetic mutations that reduce social approach or attention during development might reduce the probability that predictable aspects of the social environment are learned at all. Of course, these kinds of effects depend critically on the kinds of input that an organism receives from its environment during these social interactions. It is possible to think of the organizational effects of nonapeptide circuitry independent of its social environment, but more likely, the kinds of social experiences an organism has—and their sensitivity to those social experience—coevolved with each other. Neuropeptide systems during development may have, thus, evolved to allow organisms to plastically respond to their environment as they mature. However, by allowing for variable outcomes in adulthood, these evolvable systems also provide the raw material for evolution to act.

## Developmental Effects of AVT on Affiliation and Song Learning in the Zebra Finch

Recent experimental evidence from manipulating the nonapeptide system early in life in zebra finch provides support for the idea nonapeptides play an organizational role in on a broad suite of social behaviors (162–164). Intracranial injections of either AVT or [Manning Compound (MC), a V1aR antagonist] in hatchlings (days 2–8 post-hatching) altered social interest in the parents and conspecifics after fledging, suggesting that the nonapeptides are serving to gate a number of social approach behaviors in juvenile zebra finches (162). In addition, early life nonapeptide treatment also altered affiliative behaviors and courtship song in adult male, but not female, zebra finches. Both AVT and Control males showed an increased affiliative interest in females as they reached reproductive maturity (162). However, AVT-treated males showed less sexually motivated courting of females compared to Controls and instead formed highly affiliative pair bonds with their female partner (163). By contrast, MC males did not show the normal increase in affiliative interest in females as they reached maturity and showed only modest levels of both courtship and affiliation in their interactions with females (162, 163). Furthermore, nonapeptide treatment also altered neural activity and the expression of V1aR in the BSTm and MeA (163). Taken together, these findings suggest that AVT-injected males may have had more experience attending to social cues or a stronger association between affiliative interactions and reward compared to both MC and Control males, resulting in different approaches to reproduction.

This change in the affiliative interest in parents and conspecifics also had functional consequences for social learning. Male zebra finches injected with MC as hatchlings both showed decreased interest in their parents during development and ultimately sang a song that was a worse acoustic match to their father’s song in adulthood compared to Controls (162, 164). By contrast, AVT males showed increased affiliative interest in their parents and family and more effectively copy their father’s song (162, 164). Interestingly, affiliation with parents at 30 days post-hatch was correlated with song quality in adulthood. These data suggest that the nonapeptides may bias the motivation of developing zebra finches to attend to the behaviors of the father during development, which ultimately allows them to more accurately learn courtship song from their father. This is ultimately consistent with Syal and Finlay’s hypothesis that the nonapeptides gate complex vocal learning in song birds by altering social motivation, supporting their suggestion that the nonapeptides may play an equally critical role in language learning in humans (158).

Thus, social phenotypes may evolve via relatively simple alterations to the actions of a single nonapeptide during development. In zebra finches, AVT altered early social behaviors, potentially affecting the opportunities for social learning. However, it also affected the organization of the neural substrate underlying these social behaviors. It will likely prove impossible to disentangle the direct effects of nonapeptides on the brain during development from their indirect effects resulting from how they alter the trajectory of learning from early social experiences. Indeed, this conceptual challenge is at the heart of the nature “versus” nurture debate (4).

## Conclusion

Nevertheless, these results provide support for the idea that the actions of nonapeptides in development may play an important role in the evolution of novel social behavior. The field of evolutionary developmental biology (evo-devo) has long been concerned with how evolution shapes developmental processes to generate phenotypic novelty. However, the insights from evo-devo have rarely expanded into the social domain (165–167). Neuropeptides and hormonal systems are well-situated to play that role, given that they alter the activities of whole neural circuits. However, we are just scratching the surface in our understanding of the diversity of mechanisms which may facilitate the evolution and development of social behaviors.

Indeed, the nonapeptides are almost certainly not the only chemicals that play a role in the evolution of diverse social phenotypes. We now know of more than 100 different peptides and other signaling molecules, each of which is expressed in only a small population of neurons, and all of which signal to neurons throughout the brain via specific receptors. The endless forms of neural systems and behavior appear to be result of evolutionary changes to compartmentalization of neuropeptide signaling systems (142). However, the complex nature of diverse signaling systems suggests that they can only be fully understood by integrating research at all levels of analysis—investigating both their molecular and developmental mechanisms, as well as their adaptive significance in the life of an organism.

## Author Contributions

NB wrote the article as part of her dissertation work at Cornell University.

## Conflict of Interest Statement

The author declares that the research was conducted in the absence of any commercial or financial relationships that could be construed as a potential conflict of interest.
